# Interferon responses to norovirus infections: current and future perspectives

**DOI:** 10.1099/jgv.0.001660

**Published:** 2021-10-26

**Authors:** Aminu S. Jahun, Ian G. Goodfellow

**Affiliations:** ^1^​ Division of Virology, Department of Pathology, University of Cambridge, Addenbrooke’s Hospital, Cambridge CB2 0QQ, UK

**Keywords:** calicivirus, innate immunity, immune evasion, interferon, MDA5, norovirus, RIG-I-like receptors

## Abstract

Human noroviruses (HuNoVs) are increasingly becoming the main cause of transmissible gastroenteritis worldwide, with hundreds of thousands of deaths recorded annually. Yet, decades after their discovery, there is still no effective treatment or vaccine. Efforts aimed at developing vaccines or treatment will benefit from a greater understanding of norovirus-host interactions, including the host response to infection. In this review, we provide a concise overview of the evidence establishing the significance of type I and type III interferon (IFN) responses in the restriction of noroviruses. We also critically examine our current understanding of the molecular mechanisms of IFN induction in norovirus-infected cells, and outline the diverse strategies deployed by noroviruses to supress and/or avoid host IFN responses. It is our hope that this review will facilitate further discussion and increase interest in this area.

## Introduction

HuNoV is a highly prevalent pathogen, implicated in ~20 % of all cases of diarrhoea, causing about 677 million illnesses every year [1–3]. It spreads primarily via the faeco-oral route and infects people of all ages, although the incidence is higher in children [3, 4]. Worldwide, up to 200 000 deaths per year have been reported, with severity of symptoms increasing significantly in young children, the elderly, organ transplant recipients, and people that are immunocompromised [1, 3, 4]. An infectious dose as low as 18 virus particles is able to cause infection, and infected individuals frequently present with diarrhoea, vomiting, abdominal cramps, and occasionally fever [3–5]. There is currently no effective treatment or licensed vaccine against noroviruses, a situation not helped by our limited understanding of the biology of the virus.

As a result of the development of a number of culture systems for HuNoV and the identification of the murine norovirus model, significant progress has been made in describing the interactions between noroviruses and the host cell. Numerous cellular factors and pathways have been identified as playing critical roles in the intracellular life of noroviruses. In addition, the key role of the innate immune response in controlling norovirus infection has been uncovered. However, important questions are still being explored, including the nature of the IFN response elicited by HuNoV and to what degree components of the pathway control norovirus infections. In this review, we summarise our current understanding of the host IFN response against noroviruses, and the various described or proposed mechanisms utilised by HuNoV to evade or control the host innate response. We do not however discuss the interplay between the host interferon response and microbiota in regulating norovirus infections, as this has been recently comprehensively reviewed by Walker and Baldridge [6] and by Alwin and Karst [7].

### Biology of noroviruses

Noroviruses are positive-sense single-stranded non-enveloped RNA viruses with poly-adenylated RNA genomes ranging between 7.4–7.7 kb, encoding 3–4 open reading frames (ORFs) [5, 8, 9]. The norovirus genome contains a long 5′ ORF that encodes a polyprotein that is processed into 6–7 non-structural (NS) proteins by the viral protease, and host cell caspases in the case of MNV. A second ORF that encodes the major capsid protein VP1, and a short 3′ ORF that encodes the minor capsid protein VP2 are encoded by the 3′ half of the viral genome and are produced from a viral subgenomic RNA during viral infection [5, 10]. Murine norovirus (MNV) has a fourth ORF that overlaps the VP1 sequence and encodes an accessory protein called virulence factor 1 (VF1) [11]. Like those of picornaviruses, the genomes of noroviruses are covalently linked to a small protein called viral protein genome-linked (VPg, NS5) at the 5′ end, involved in virus replication and unlike its picornavirus counterpart, is essential for viral translation [5, 8]. The NS1/2, NS3 (viral NTPase), and NS4 proteins are thought to play central roles in the formation of viral replication complexes, the NS5 protein (VPg) mediates translation of viral proteins, and the NS6 and NS7 proteins are the viral protease and RNA-dependent RNA polymerase (RdRp), respectively [9, 12]. The VP1 protein forms the bulk of the icosahedral viral capsid, being arranged in 90 dimers. In addition to representing a minor component of the capsid [5] that, based on work with the closely related feline calicivirus, may form a pore for viral genome entry [13], the VP2 protein has also been implicated in the regulation of host adaptive immune responses by manipulating the surface expression of proteins required for antigen presentation in a strain-specific manner [14, 15].

Due to the absence of a robust cell culture system to grow HuNoV in the past, much of what is known about norovirus replication came from studies on closely related viruses including MNV [9, 16]. Cell entry during an infection with noroviruses is thought to occur by receptor-mediated dynamin II- and cholesterol-dependent endocytosis, followed by uncoating to release the VPg-linked viral genome into the cytosol [5, 17]. Translation of the viral genome occurs in a VPg-dependent manner, and the viral polyprotein is then cleaved into mature non-structural proteins [16]. The non-structural proteins recruit host membranes to form peri-nuclear replication complexes, and the viral RdRp uses VPg as a protein primer for genome replication, although some RNA synthesis can also occur *de novo* [16]. The current model for viral replication suggests that *de novo* transcription by the RdRp produces a negative-strand RNA that serves as a template for both the genomic RNA and a VPg-linked subgenomic RNA that encodes the VP1 and VP2 proteins (and VF1 in MNV). Infected cells subsequently undergo apoptosis to release mature virions although recent evidence would also implicate other non-lytic processes in the release of noroviruses [18].

Both acute and persistent strains of MNV were shown to be able to infect macrophages and dendritic cells *in vitro* early on after their discovery*,* although their *in vivo* tropism was not known at the time [9, 19]. The recent description of the proteinaceous receptor for MNV [20, 21], coupled with advances in *in situ* hybridisation assays allowed for the subsequent discovery of the *in vivo* tropism of MNV to myeloid cells, lymphocytes and tuft cells [22–24]. HuNoV was recently shown to infect a B-cell cell line (BJABs) and the enterocyte component of the human intestinal enteroids *in vitro* [25–27]. HuNoV *in vivo* tropism is however not clear, although viral antigens have been detected in the intestinal epithelial cells (IECs) of infected gnotobiotic pigs, lamina propria of a biopsy sample from an infected person, and dendritic cells of an infected chimpanzee (reviewed by Karst *et al*. [9]).

### Innate immune recognition of noroviruses in infected hosts

Innate immunity encompasses an elaborate system of physical and chemical barriers, secreted and membrane proteins, as well as a myriad of effector cells that provide rapid non-specific protection from an invading pathogen. The IFN response pathway is a central component of this system, and begins with detection of pathogen-associated molecular patterns (PAMPs) by a diverse network of host receptors, leading to production of IFNs and generation of an antiviral state in affected cells (reviewed by Ingle *et al*. [28], Ivashkiv and Donlin [29], Lazear *et al*. [30], and Hoffmann *et al*. [31]). IFNs induce expression of IFN-stimulated genes (ISGs) that facilitate the resistance of host cells to viruses, activate immune cells recruited to the sites of infection and upregulate factors required for activation of adaptive immunity, all of which makes them critical in the control of viral infections (reviewed by Schneider *et al*. [32], and Schoggins [33]). For this reason, and on account of the co-evolution of hosts and pathogens, any virus that is able to infect a host is also likely to have evolved mechanisms of counteracting IFN responses [31, 34].

The retinoic acid-inducible gene 1 (RIG-I)-like receptors, RIG-I and myeloma differentiation-associated protein 5 (MDA5), are implicated in the detection of the presence of most RNA viruses in an infected host cell (recently reviewed by Rehwinkel and Gack [35] and by Carty *et al*. [36]). These pattern recognition receptors (PRRs) typically sense viral replication intermediates in the cytoplasm. RIG-I is thought to sense uncapped 5′-tri- and di-phosphorylated single-stranded or short double-stranded RNA, while MDA5 detects longer double-stranded RNA, all typically seen in viral genomes or present as viral replication intermediates [35–39]. Other PRRs that can detect RNA viruses include toll-like receptors (TLRs) such as TLR2 (detects viral capsids at cell surfaces [36, 40]), TLR3 (detects dsRNA [36, 41]), and TLR7/8 (detect ssRNA in endosomes [36, 41]), as well as nucleotide-binding oligomerisation domain (NOD)-like receptors such as NLRP6 (detects cytosolic dsRNA [42]). Recently, the DNA receptor cyclic GMP-AMP synthase (cGAS) was shown to detect leaked mitochondrial DNA potentially resulting from either mitochondrial damage from membrane recruitment [43, 44] or mitochondrial leakage downstream of IL-1β signalling [45] in RNA virus-infected cells. Activation of these PRRs leads to recruitment of adapter proteins, activation of downstream kinases and transcription factors, and eventually expression of IFNs.

**Fig. 1.**
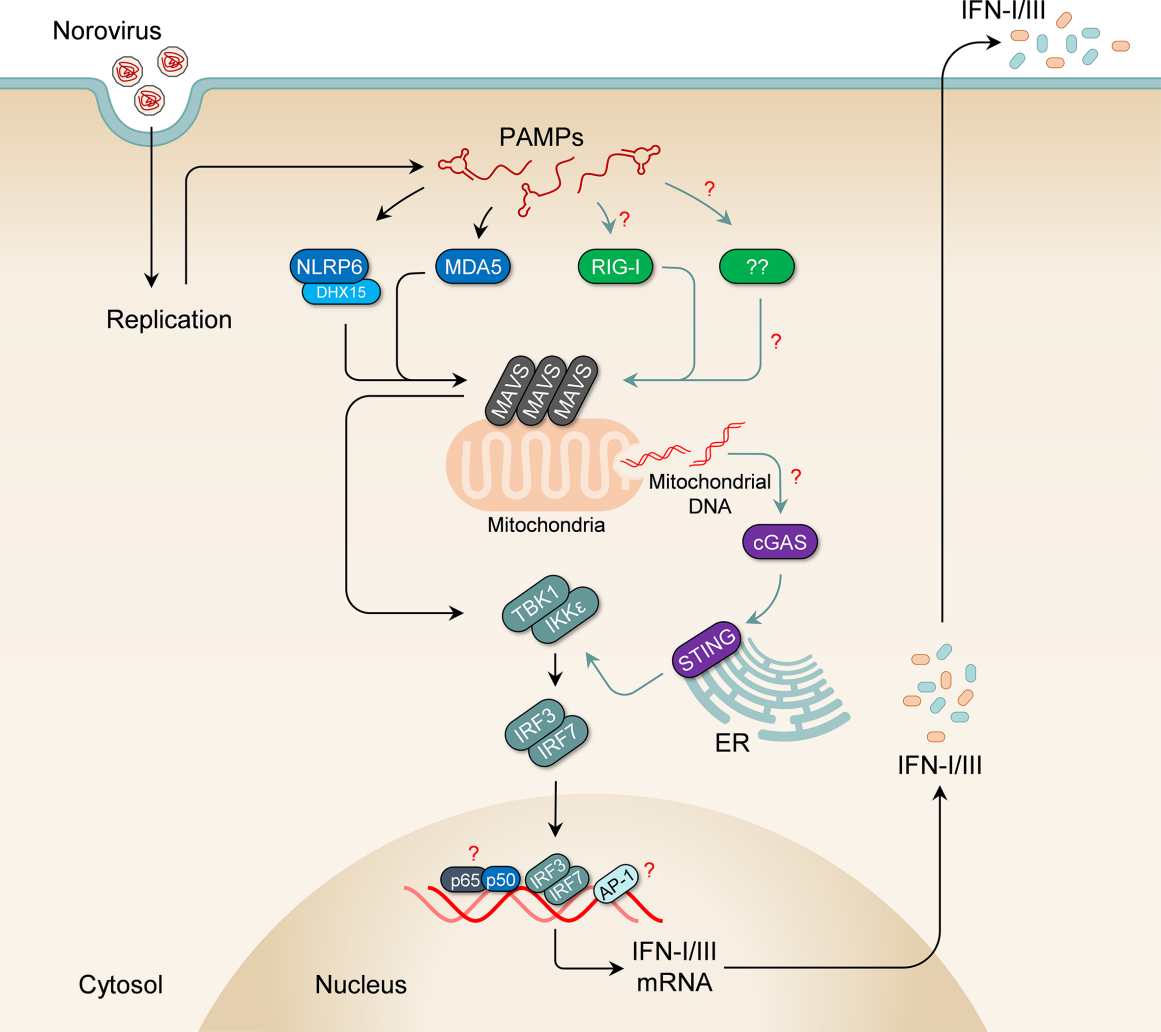
Mechanism of IFN induction in norovirus-infected cells. Pathogen-associated molecular patterns (PAMPs), generated from virus replication, are thought to be detected by MDA5 and NLRP6, leading to activation of MAVS at mitochondria and peroxisomes. Activated MAVS in turn activates downstream kinases, TBK1 and IKKε, which recruit and phosphorylate IRF3 and IRF7. This results in their dimerization and translocation into the nucleus, where they induce expression of type I and type III interferons. The interferons produced are then released to act on cells in an autocrine and paracrine manner. Although clear experimental evidence is lacking, it is likely that additional pattern recognition receptors, such as RIG-I, cGAS and/or others, contribute to the sensing of norovirus PAMPs. For example, RIG-I is able to detect transcripts generated by the norovirus polymerase when over-expressed in cells, and cGAS can sense leaked mitochondrial DNA that results from IL-1β signalling.

MDA5 was shown to play a central role in the innate immune response to both acute and persistent strains of MNV (Fig. 1) [46, 47]. Near-baseline levels of IFN-α were seen in bone marrow-derived dendritic cells (BMDCs) from MDA5-deficient (*Ifih1* ^-/-^) mice following infection with an acute strain of MNV (CW3) compared to the wild-type, with a significant increase in viral titres seen in the spleen, mesenteric lymph nodes (MLN) and proximal intestines. The role of MDA5 in restricting MNV replication was shown to be limited to the IFN response pathway, as similar levels of viral inhibition were obtained from wild-type and *Ifih1* ^-/-^ cells following pre-treatment with IFN-α. Surprisingly, while higher viral titres were seen in *Ifih1* ^-/-^ mice infected with a persistent strain of MNV (CR6) compared to wild-type mice, there was no difference in wild-type and *Ifih1* ^-/-^ BMDCs [47]. The authors speculate that the inability of BMDCs to sense type III IFNs could account for this disparity. Given that the evidence for the inability of mouse BMDCs to respond to type III IFNs is conflicting [48–50], it could also indicate a strain-specific role for other PRRs. Indeed, the increase in viral titres observed in MDA5 knockout mice and BMDCs infected with acute strains of MNV are only moderate compared to those seen in *Stat1* knockout mice and cells, respectively. Additionally, infection in MDA5 knockout mice is not lethal, in contrast to that in *Stat1* knockout mice [46, 51], further suggesting the presence of other receptors that contribute to the restriction of viral replication (reviewed by Karst [52]).

NLRP6 was also shown to contribute to cytosolic detection of MNV, likely in a manner dependent on the RNA helicase DHX15 [42]. Increased – though modest – viral titres were obtained in intestinal epithelial cells, spleen and faeces from *Nlrp6* ^-/-^ mice compared to wild-type mice, with faecal shedding persisting beyond 8 days post-infection. In this context, NLRP6 acts like a typical RIG-I-like receptor, interacting with MAVS, and using DHX15 as a co-receptor [53]. The authors demonstrated an inverse relationship in the expression of MDA5 and NLRP6 in intestinal epithelial cells and myeloid cells in the gut, with higher levels of MDA5 in myeloid cells and NLRP6 in intestinal epithelial cells, and posit that NLRP6 likely complements MDA5 detection of viruses in epithelial cells.

TLR3, TLR7, and presumably TLR8 (via Myd88) do not appear to play any role in IFN responses to noroviruses *in vitro*, although a marginal increase in viral titres was observed in the MLNs of *Tlr3* ^-/-^ mice [46, 47]. Interestingly, no study published to date has examined MNV infection in RIG-I-deficient (*Ddx58* ^-/-^) cells. It was previously shown that RIG-I, like MDA5, can detect RNA transcripts made by the MNV polymerase when over-expressed in HEK293T and Huh-7 cells [54]. However, in a recent study looking at RIG-I inhibition by a bacterial quorum-sensing molecule, it was shown that while treating cells with the molecule led to a moderate increase in SeV titres in HEK293T cells, it did not appear to affect MNV titres in RAW264.7 cells [55]. It is not clear whether there was any effect on IFN induction by the MNV infection or if the dose of the inhibitor used was sufficient in these experiments. Also, no statistically significant difference was observed in viral replication following transfection of HuNoV RNA in Huh-7 and the RIG-I-deficient Huh-7.5 cells, although norovirus replication in these cell lines did not induce an IFN response [56, 57]. In contrast, HuNoV replication in human gastric tumour cells does result in IFN activation and long-term maintenance of a HuNoV replicon requires the suppression of type III interferon responses [58]. Whether RIG-I contributes to restriction of noroviruses is therefore still unclear.

The phenotypic differences between MNV-infected MDA5 and STAT1 knockout cells are unlikely to be explained solely by the contribution of NLRP6 in norovirus detection, indicating a potential role for other receptors. While the presence of VPg was always thought to protect the viral genome from RIG-I sensing [46, 59], there is currently insufficient experimental evidence to rule it out as a sensor of noroviruses. Moreover, recent studies on picornaviruses and on the Tulane virus show that RIG-I can still detect viruses that have VPg-linked RNA genomes [60–63]. Other PRRs can also potentially participate in the recognition of noroviruses, including the DNA sensor cGAS for example, which was recently shown to indirectly recognise infection with dengue viruses by sensing leaked mitochondrial DNA [43, 45]. The release of mitochondrial DNA into the cytosol was shown to occur downstream of IL-1β [45], a proinflammatory cytokine abundantly secreted by MNV-infected cells [64]. While this pathway may not be activated in cells infected with many other RNA viruses [65], whether it is activated in norovirus-infected cells remains to be explored. A recent study also suggested that TLR2, a capsid-sensing PRR [40] expressed on cell surfaces, can bind HuNoV virus-like particles [66], although it is not clear if this interaction leads to an IFN response. These examples and others highlight the need for more work in this area.

Downstream of the PRRs, MAVS, IRF3 and IRF7 have all been shown to play important roles in induction of IFN following infection with MNV [47, 67, 68]. HOIL1 (Heme-oxidized IRP2 ubiquitin ligase 1), a component of the linear ubiquitin assembly complex (LUBAC), was also recently shown to contribute to IFN induction in MNV-infected mice, and the authors speculated that it likely acts downstream of MDA5 [47]. Like in MDA5-deficient mice, HOIL1-deficient (*Rbck1^-/-^*) mice infected with a persistent strain of MNV (CR6) had higher viral titres in the stool, colon, ileum and MLN, and no difference in viral titres in BMDMs despite a significant reduction in IFN induction. However, direct mechanistic evidence connecting MDA5 and HOIL1 remains to be uncovered, and the levels of SHARPIN (SHANK-associated RH31 domain-interacting protein) were also consistently reduced in the cells used indicating possible alternative explanations for the phenotypes observed. Moreover, the LUBAC complex has previously been shown to inhibit RLR signalling, while also activating NEMO and promoting IRF3-dependent apoptosis [69–71]. Nevertheless, these studies indicate a complex role for linear ubiquitination in controlling infections with RNA viruses and more work is thus required to understand it.

### Potential ligands detected in norovirus-infected cells

**Fig. 2.**
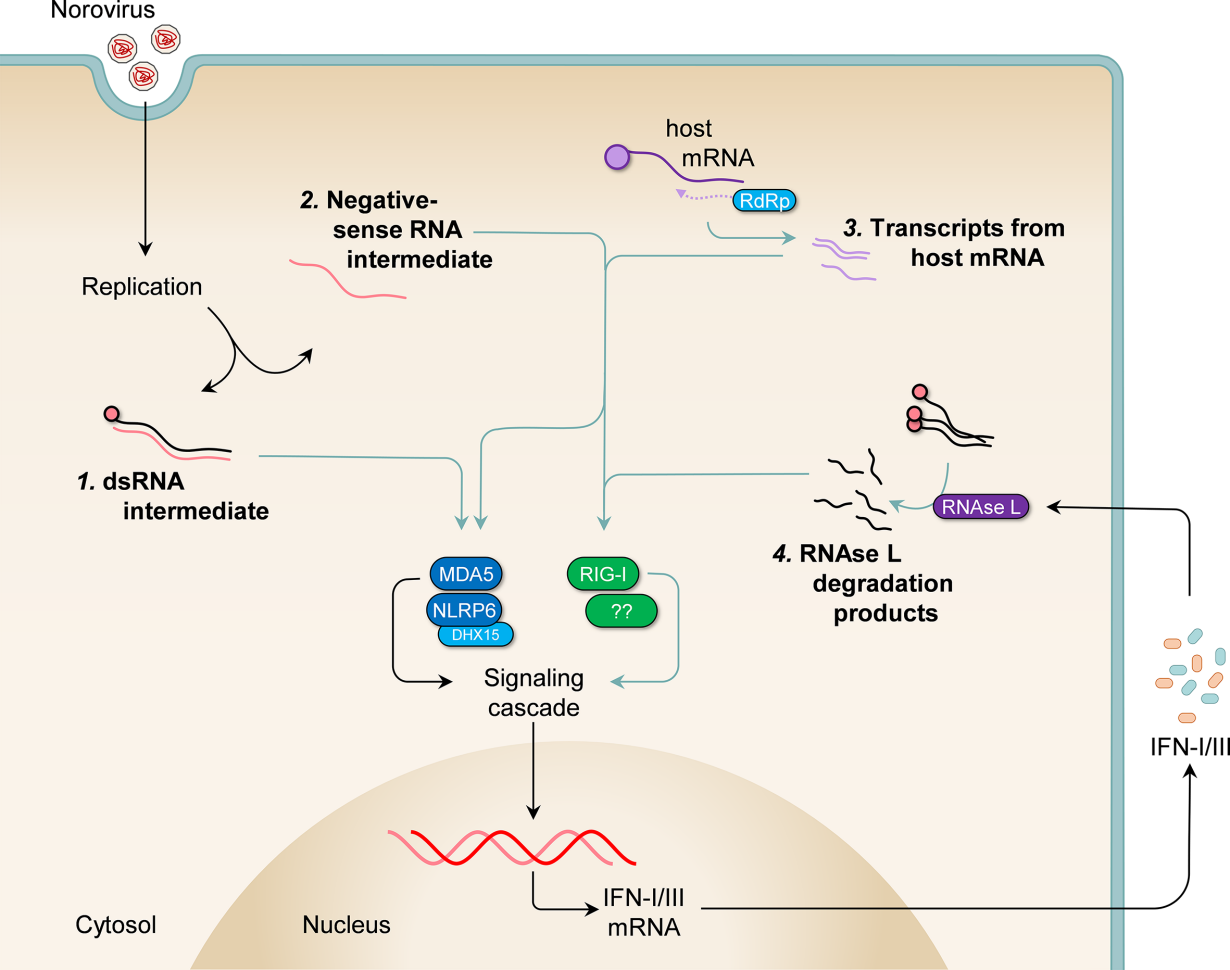
Possible PAMPs detected in norovirus-infected cells. Replication of noroviruses in the host cell likely generates PAMPs detected by intracellular PRRs. Although the identities of these PAMPs are yet to be determined, it is possible they include the double-stranded RNA intermediate composed of the VPg-linked positive-strand RNA and the *de novo*-synthesized negative-strand RNA, which may be detected by MDA5 (1). Single-stranded negative sense RNA intermediates that are likely not VPg-linked, produced during replication, could also be potential ligands for other host PRRs such as RIG-I (**2**). Additionally, MDA5, and perhaps RIG-I, may recognise RNA species transcribed by the viral polymerase from host RNA templates (**3**). And lastly, it is possible that MDA5 or RIG-I can detect RNA fragments produced by RNAse L digestion of host and viral RNA in norovirus-infected cells (**4**).

Work on optimising vaccine strategies targeting noroviruses may benefit from knowledge of specific PAMPs that can be included as adjuvants. However, there is currently a lack of data regarding the precise PAMPs detected in norovirus-infected cells. The MNV genome is released into the cytosol less than an hour post-infection [5] and IFN transcripts are upregulated as early as 4 hours after infection [72]. While it is possible that the genomes from incoming viruses are detected by sensors such as MDA5 in the cytosol, this is unlikely as proteinase K treatment prior to transfection of viral RNA or gamma irradiation pre-infection abrogates IFN induction [46], suggesting that viral replication is required for the generation of the norovirus PAMPs detected by the PRRs (Fig. 2). This is also true for HuNoV [73] and is similar to other RNA viruses such as influenza A virus, vesicular stomatitis virus, and Semliki Forest virus [74–76].

The most likely candidate MDA5 ligand includes the double-stranded RNA intermediate composed of the VPg-linked positive-strand RNA and the *de novo*-synthesized negative-strand RNA [16]. Additionally, it has been shown that the MNV polymerase can transcribe RNA species that are detected by both MDA5 and RIG-I, but not TLR3 [54, 77], thus single-stranded negative sense RNA intermediates produced during replication and which likely lack VPg linkage, could therefore be potential ligands for RIG-I. MDA5, and perhaps RIG-I, may also recognise RNA species transcribed by the viral polymerase from host RNA templates as demonstrated in Semliki Forest Virus-infected cells – an Alphavirus that has a positive-sense single-stranded RNA viral genome akin to noroviruses [78]. And lastly, it is possible that MDA5 or RIG-I can detect RNA fragments produced by RNAse L digestion of host and viral RNA in norovirus-infected cells, as was shown for other RNA viruses [79–82], especially in light of previous work that indicated a potential role for RNAse L in restricting MNV replication in an IFN-γ-dependent manner [83].

### IFN induction and release during norovirus infections

Consistent induction of type I and type III IFNs is seen following infection with MNV both *in vivo* and *in vitro* (Table 1) [46, 47, 72, 84–86]. In mice, IFN-β is detected in intestinal homogenates as early as 12 h following per-oral inoculation, and in the serum within 24 h of infection [86]. *In vitro*, while an increase in transcripts is seen early during infection, IFN release appears to be temporally different in cell lines compared to primary cells, with IFN-β secretion seen within 4 h of infection in BMDMs, and 20 h in the RAW264.7 macrophage cell line [72]. This unexplained delay in IFN release is potentially responsible for the higher viral titres seen in macrophage cell lines compared to primary macrophages and dendritic cells [72].

**Table 1.** Do noroviruses induce interferons?

s/n	Virus	Model system	IFN induction	IFN subtype	References
1.	MNV	Mice	Yes	IFN-β, IFN-ʎ	[47, 86, 89, 97]
		BMDMs	Yes	IFN-β	[72]
		BMDCs	Yes	IFN-α, IFN-β, IFN-ʎ	[46, 47, 89]
		ER-HoxB8 DCs	Yes	IFN-β, IFN-ʎ	[47]
		RAW264.7	Yes	IFN-β	[11, 72]
		M2C-CD300lf	Yes	IFN-α, IFN-β, IFN-ʎ	[23]
2.	HuNoV	Human volunteers	Yes	IFN-α, IFN-γ	[91]
		Gnotobiotic pigs	Yes	IFN-α, IFN-γ	[93]
		Gnotobiotic calves	Yes	IFN-γ	[131]
		Zebra fish	Yes	Not specified	[132]
		Organoids	Yes	IFN-ʎ	[73, 94, 95]
		Huh7	Unlikely	Not specified	[57]
		HEK293FT	No	Not specified	[90]
		HGT-NV	Likely	Not specified	[58]

There also appears to be a strain-specific difference in IFN-β induction following MNV infection *in vitro*, even in the absence of differences in growth kinetics. The persistent S99 strain, for example, elicits a significantly attenuated induction compared to the acute MNV1.CW3 virus [87], and the persistent MNV3 induces considerably higher levels compared to the acute MNV1.CW1 [88]. A variant of MNV3 that does not persist also showed higher IFN-β secretion compared to MNV1.CW3 [14]. Interestingly, while both the persistent CR6 and the acute CW3 MNV strains induce type I IFNs in primary cells [46, 47], CR6 does not induce type I or type III IFNs in infected mice [89]. Taken together, these studies suggest that infection with MNV perhaps triggers a differential strain-specific, and possibly cell type-specific, IFN response.

In contrast to MNV, the nature of the IFN response in HuNoV-infected host cells has been an open question for decades (Table 1). In early experiments, cell culture supernatant from poly (I:C)-transfected cells was able to inhibit replication of HuNoV in Huh-7 cells, but not that from norovirus RNA-transfected cells [57], suggesting that transfection of HuNoV RNA into cells does not induce IFNs. Similar results were obtained in 293FT cells, where there was no IFN induction following transfection of HuNoV RNA compared to control RNA [90]. Infection with Sendai virus (SeV) or secondary transfection of poly (I:C) in these norovirus RNA-transfected 293FT cells led to a robust induction of IFN-β, indicating that replication of HuNoV did not interfere with the IFN response pathway itself. Additionally, siRNA depletion of MAVS and IRF3, proteins mediating central roles in the IFN induction pathway, did not affect viral replication, further suggesting lack of innate immune detection of human noroviral presence in these cells. This contrasts with data from human studies where serum levels of IFN-α2, IFN-γ and other cytokines were increased following infection with HuNoV [91, 92]. It also contrasts with work in gnotobiotic pigs, where an increase in IFN-α and IFN-γ were seen as early as 24–48 h after infection, with a second peak for IFN-α seen after 10 days in the serum and gut of infected animals [93]. Moreover, more recent studies by our group and others suggest a considerable role for endogenous IFNs in controlling replication in human intestinal organoids and in a HuNoV replicon-containing cell line [58, 73, 94]. One caveat with the earlier *in vitro* experiments is that in both the Huh-7 and 293FT studies, purified RNA was used from stools of norovirus-infected humans which may contain other contaminating RNA. Also, levels of viral replication seen in this system is very low, with less than 0.1 % of cells in a transfected culture showing evidence of replication. Nevertheless, these preliminary studies hitherto offered the only available accounts of IFN responses to HuNoV in cell lines, or their lack thereof, in the absence of a robust culture system, and highlight the need for more work in this area.

Emerging evidence from *in vitro* studies performed in human intestinal organoids suggest that HuNoV infection induces abundant expression of type III IFNs, but almost no type I [73, 95]. Given that human challenge studies have shown expression of type I IFNs during infection [91], this preferential induction of type III IFNs is possibly an artefact of the organoid model, for a number of reasons. First, all the recent data showing preferential expression of type III IFNs following HuNoV infection came from three organoid lines only [73, 95], and although unlikely, whatever phenotype is demonstrated could be an idiosyncrasy of these particular lines. Secondly, intestinal epithelial cells, the primary targets of HuNoV in organoids, do preferentially express type III IFNs on account of their abundance of peroxisomes [96], and indeed organoids infected with HuNoV produced type I IFNs at fairly similar levels as those treated with poly (I:C) [73]. Thirdly, intestinal organoids used for infection with HuNoV are typically differentiated into monolayers of cells that are a heterogenous population [27, 73] of which the enterocytes are the primary cell type in the culture that are susceptible to infection [27]. It is thus possible that a marginal, but potentially potent, type I IFN expression in infected cells is masked by its lack thereof in the non-susceptible population of cells in the culture. This hypothesis is in keeping with the considerable increase in viral titres seen in type I IFN receptor-deleted organoid lines compared to wild-type lines [73]. Therefore, while these early data from organoids are illuminating, the picture is still far from complete.

### Restriction of norovirus replication by IFNs

Data from both *in vivo* and *in vitro* studies have established the capacity of IFNs to restrict replication of MNV. First, MNV infection in wild-type mice is largely asymptomatic, in contrast to *Stat1* ^-/-^ or types I and II IFN receptors-deficient (IFNαβγR^-/-^) mice, in which infection is accompanied by a considerable increase in viral RNA and causes severe symptoms, significant multi-organ pathology, and death in all infected mice within 2 weeks of infection [51, 86]. This increased susceptibility in IFNαβγR^-/-^ mice can be reversed by introduction of an IFN-λ-expressing plasmid [97]. Selective knockout of *Ifnar1* in dendritic cells also allows an otherwise acute strain of MNV (CW3) to persist, despite the presence of a functional adaptive immune system [98]. Secondly, treatment of MNV-infected cells with recombinant IFN-β or IFN-λ inhibits viral replication [23, 83, 99]. Similarly, TLR7 agonists and interferogenic plant extracts (Schizonepeta tenuifolia Briquet) significantly inhibit MNV replication by promoting IFN induction [100, 101]. *In vivo* experiments in mice showed a context-dependent differential requirement of IFN subtypes in which type I IFNs protect against systemic spread via immune cells while type III IFNs restrict enteric persistence [89].

While there are no data on the restriction of HuNoV replication by IFNs in human subjects, infected gnotobiotic pigs showed decreased faecal shedding following treatment with IFN-α [102]. We have also observed that long-term replication of a HuNoV replicon in intestinal epithelial cells is accompanied by the epigenetic suppression of the IFN-lambda receptor expression [58]. We hypothesise that this is not an active process induced by HuNoV replication, but rather occurred spontaneously in culture, and is more likely a reflection of a selective advantage that cells with reduced IFN receptor expression have. In such an example, cells with the reduced type III IFN receptor would respond less well to type III IFN induced in the culture, allowing higher levels of replicon to accumulate, resulting in greater resistance to the antibiotic used to select for the maintenance of the replicon in the cells. Additionally, our group and others have demonstrated that treatment of HuNoV replicon-harbouring HG23 cells or human intestinal organoids with different IFN sub-types also results in a reduction in viral genomes in a dose dependent-manner [73, 94, 103–105], and treatment of Huh-7 cells with cell culture supernatant from poly (I:C)-transfected cells inhibited replication of HuNoV RNA [57]. Overall, these studies show the capacity of IFNs to inhibit replication of HuNoV, as it does MNV.

The specific ISGs responsible for inhibiting norovirus replication are not all known. Pre-treatment of cells with recombinant IFN-β or IFN-γ was shown to inhibit translation of MNV proteins without affecting viral genome integrity [83]. This inhibition was shown to be independent of PKR and RNAse L for the IFN-β-pre-treated cells, but not in IFN-γ-pre-treated cells, indicating the presence of another ISG(s) that inhibits viral translation. Several ISGs have since been shown to generally inhibit translation of viral proteins via disparate mechanisms (reviewed by Li *et al*. [106]), but their functions have not been looked at in the context of a norovirus infection. ISG15 is among the few ISGs clearly implicated in restricting norovirus replication. Higher viral titres were obtained from IFN-α-treated IS15-deficient bone marrow-derived macrophages (BMDMs) compared to wild-type cells, indicating a role for ISG15 in IFN-dependent control of MNV replication [99]. This function was shown to be at the level of viral entry or uncoating, as replication in MEFs transfected with the MNV RNA was not affected by the absence of ISG15. A recent study, using a CRISPR activation screen in a human cell line to look for host restriction factors of MNV, demonstrated antiviral activities of a number of ISGs on MNV infection, including MX1 and TRIM7, although using cell survival as the primary readout in the study likely precluded the numerous ISGs that themselves mediate cell death [107]. Other ISGs shown to counteract norovirus replication are mostly involved in the IFN response and antigen presentation pathways, and include NLRP6, IRF1, IRF7, IFN-λs, STAT1, IFIT1, MHCII, and β2M [68, 108]. It should be noted that MNV has been shown to inhibit ISG translation through different independent mechanisms [84], leading to very low ISG levels in infected cells [84, 99].

### Counteraction of IFN responses by noroviruses

**Fig. 3.**
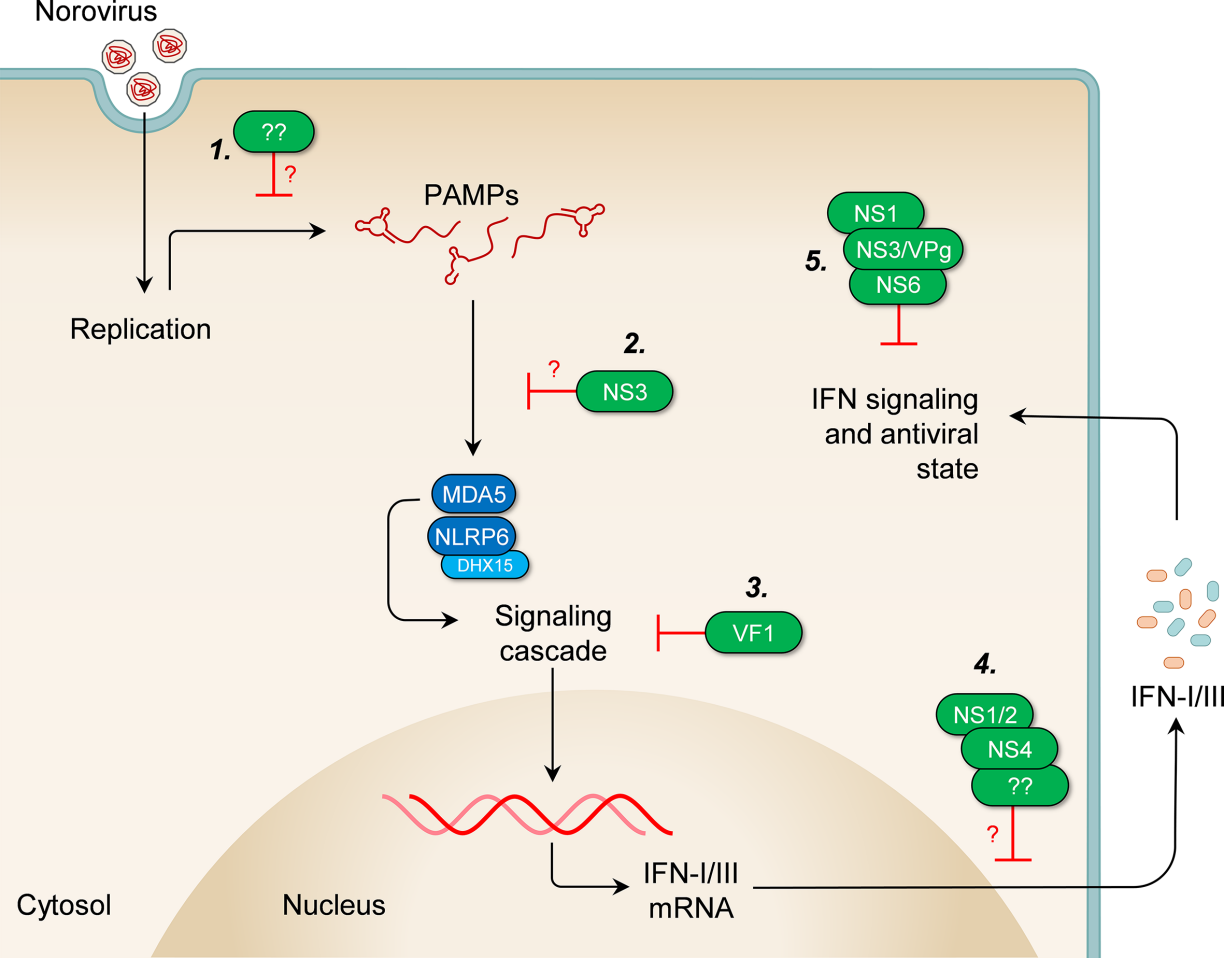
Evasion of IFN responses by noroviruses. Several strategies have been demonstrated or proposed through which noroviruses counteract the different stages of the host IFN response. (**1**) *Avoidance of detection:* certain strains of mouse and human noroviruses appear to induce very low levels of IFNs in infected cells, possibly by avoiding detection of viral ligands by host receptors, through yet unknown mechanisms. (**2**) *Impairment of PRR functions:* the NS3 protein may inhibit IFN induction by redistributing host GEF-H1, thus perhaps impeding the functions of host receptors of viral ligands. (**3**) *Inhibition of the signalling cascade:* the VF1 accessory protein inhibits IFN induction downstream of TBK1, through a yet unknown mechanism. (**4**) *Obstruction of IFN release:* the NS1/2 and NS4 proteins promote Golgi disassembly and disruption of ER-Golgi trafficking, thereby potentially impairing cellular secretory pathways utilized for release of IFNs. Other unknown factors in some strains of norovirus may also impair IFN release. (**5**) *Disruption of IFN signalling and the antiviral state:* the NS1 protein mediates persistence in type I IFN-resistant IECs and neutralises type III IFN signalling. The NS6 protein cleaves PABP and the VPg and/or NS3 proteins sequester G3BP1 in replication complexes, thereby potentially contributing to the impairment of translation of ISGs.

As our understanding of innate sensing and restriction of norovirus infections has grown, so too has our knowledge of the wide range of strategies the viruses use to evade immune responses (Fig. 3, Table 2). The VF1 protein is the first norovirus protein shown to antagonize the IFN response [11, 14, 88]. It is a small 213-amino acid protein encoded by an alternate open reading frame overlapping the VP1 sequence. It is a mitochondrial protein, present only in MNV, and is not encoded by HuNoV [11, 88]. When RAW264.7 cells are infected with MNV1 M1, a VF1-deleted mutant, they show an increased induction of IFN-β and an impaired ability to activate apoptotic pathways compared to those infected with the wild-type virus [11]. Deletion of VF1 exacts a fitness cost on the virus in RAW264.7 cells and the M1 mutant reverted to wild-type virus after three passages. Although the mechanism is not clear, VF1 inhibited IFN induction after over-expression of RIG-I, MDA5, MAVS, and TBK1, indicating that it likely acts downstream of TBK1 activation [11, 14]. Mice infected with MNV1 M1 show decreased viral titres on days 3 and 5 in all tissues tested, including MLNs, spleen, liver, kidney, intestine, heart, lung and faeces, compared to those infected with the wild-type virus [11]. Taken together, these findings demonstrate a clear strategy by MNV to counteract IFN responses through expression of an accessory protein, although the exact mechanism and target of this action have not been determined.

**Table 2.** Antagonism of host IFN responses by noroviruses

s/n	Protein	Virus	Mechanism	Outcome	References
1.	NS1	MNV.CR6	Unknown	Mediates persistence in type I IFN-resistant IECs	[23, 113, 133]
2.	NS1	MNV.CR6	Unknown	Neutralises type III IFN signalling in IECs	[23, 113]
3.	NS1/2 and NS4	HuNoV GI.1, HuNoV GII.3, HuNoV GII.4, HuNoV GII.6, MNV1.CW3, MNV.CR6	Golgi disassembly and disruption of ER-Golgi trafficking	May impair cellular secretory pathways utilized for release of IFNs	[123–127]
4.	NS3	MNV1.CW1	Intracellular redistribution of GEF-H1	May prevent potential detection of viral PAMPs by GEF-H1	[130]
5.	NS3 or VPg	MNV1.CW1	Sequestration of G3BP1 and G3BP2 inside replication complexes	May further impair translation of ISGs	[117–119]
6.	NS6	MNV1.CW1	PABP cleavage	Inhibits translation of ISGs	[84]
7.	NS7	MNV1.CW1	GBP2 binding	Inhibits type II IFN-dependent antiviral responses	[134]
8.	VF1	MNV1.CW1, MNV3	Unknown	Inhibits type I IFN induction	[11, 14, 88]
9.	Activated caspases	MNV1.CW1	eIF4E cleavage	Inhibits translation of ISGs	[84]
10.	Unknown	HuNoV GI.1	Possible sequestration of viral genomes within replication complexes	Likely prevents detection of viral PAMPs	[90]
11.	Unknown	MNV.S99	Unknown	Attenuated type I IFN release	[87]

Although the mechanism still remains to be fully elucidated, the MNV NS1 protein mediates persistence of the CR6 strain of MNV in Tuft cells, a subset of mouse IECs [23, 24, 109–111], and may therefore also contribute to the avoidance or control of the innate immune response. Replacing the CR6 NS1 with that of CW3, the acute strain, led to clearance of the persistent virus, while replacing the CW3 NS1 with that of CR6 led to persistence of the acute strain in IECs [23]. Furthermore, the CR6 virus expressing CW3 NS1 was shown to persist in the absence of the IFNLRα [22]. While the IECs express the IFNLRα and respond readily to type III IFNs, they show a minimal response to type I IFNs [112], and persistence in them therefore allows for escape from type I IFN responses. Additionally, infection with the CR6 strain impairs expression of type III IFN-dependent genes in a manner dependent on NS1 [113], indicating potential neutralisation of type III IFN responses by the CR6 NS1.

Very low levels of ISG proteins are seen in MNV-infected cells [84, 99], as the virus inhibits ISG translation via disparate mechanisms. First, the viral protease was shown to cause cleavage of poly A-binding protein PABP, required for cap-dependent, but not VPg-dependent, translation [84, 114]. Cleavage occurs at position Q440 of PABP and allows for a disruption of host translation while translation of viral proteins occurs unimpeded. Secondly, MNV infection triggers apoptosis and caspase-dependent cleavage of eIF4E and other translation initiation factors [84]. While the specific role of eIF4E in the replication of MNV is not clear, its depletion affects cap-dependent translation of host proteins, but does not seem to affect translation of viral proteins [115, 116]. In addition, our group and others recently showed that Ras-GTPase activating SH3 domain binding protein 1 (G3BP1), a stress granule assembly factor, is sequestered within viral replication complexes in MNV-infected cells [117–119], likely via an interaction with the viral NS3 [119] or VPg [118]. G3BP1 is important for translation of ISGs in virus-infected cells [120–122], and its depletion from the cytosol potentially further impairs the ability of the cells to establish the antiviral state.

Other strategies deployed by noroviruses to evade IFN responses have been proposed. First, the HuNoV non-structural proteins NS1/2 (p48) and NS4 (p22) were implicated in Golgi disassembly and disruption of ER-Golgi trafficking of cellular proteins and thereby potentially impairing cellular secretory pathways utilized for release of IFNs [123–127] (reviewed by Roth and Karst [128]). The mechanism for this activity is still under investigation, and its direct effect on IFN responses in the context of a viral infection remains to be tested [128]. Secondly, considering that no evidence of IFN induction was observed in Huh-7 and 293FT cells harbouring HuNoV RNA, it has been suggested that the viral genomes could be sequestered within replication complexes and away from the RLRs [57, 90]. This is however becoming increasingly uncertain since this observation was made in culture systems where replication occurs in only a tiny fraction of cells in the culture, and contrasts with data from other models of norovirus infection. Thirdly, guanine nucleotide exchange factor-H1 (GEF-H1) was recently shown to promote IFN induction downstream of RIG-I and MDA-5 [129]. Although depletion of GEF-H1 using siRNA did not affect IFN levels or viral titres following MNV infection, its intracellular distribution was changed in cells expressing MNV NS3 and it was found to localise to the viral replication complex in infected cells, suggesting that the NS3 protein could be interfering with IFN induction by targeting the protein [130]. And lastly, the persistent S99 strain of MNV displayed a significantly attenuated IFN response in a mouse macrophage cell line compared to the CW3 strain, despite sharing similar growth kinetics, suggesting the presence of a yet unknown strain-specific IFN response evasion mechanism [87]. Further work is warranted to confirm these, and other potential strategies employed by noroviruses to evade IFN responses.

### Concluding remarks and future perspectives

The IFN response is the first line of defence against viruses, including noroviruses, and is a major determinant of infection. Despite significant progress in the past towards understanding this host response to noroviruses, many questions still remain. For instance, while MDA5 has been established as a bona fide PRR in MNV-infected cells, the contribution of other PRR is expected but yet undefined, considering that the increase in viral titres seen after STAT1 depletion are considerably higher than that seen after MDA5 depletion. Current available data provide support for other receptors such as RIG-I, and warrant future examination of their roles in norovirus restriction and of the cognate PAMPs recognised during infection with noroviruses. Additionally, although regulation of IFN responses by the NS1 and VF1 proteins, among others, have been demonstrated, the mechanisms by which they effect this are still unclear. Future studies are needed to further understand the function of these viral proteins in subverting IFN signalling, as well as other potential strategies deployed by noroviruses to evade host innate immune responses.

IFN responses constitute a key component of the antiviral arsenal of an infected host. A significant limitation in studying host responses against HuNoV has been the lack of a robust cell culture system. It is expected that recent advances in *in vitro* culture of HuNoV will allow us to answer the questions raised here and many others regarding the biology and pathogenesis of norovirus infections in humans. Future research focussing on understanding the molecular mechanism and regulation of IFN responses to HuNoV will in turn also likely facilitate further improvements on the current available culture systems for the virus.
